# Sequence Blockiness Controls the Structure of Polyampholyte Necklaces

**DOI:** 10.1021/acsmacrolett.1c00318

**Published:** 2021-07-28

**Authors:** Artem M. Rumyantsev, Albert Johner, Juan J. de Pablo

**Affiliations:** † Pritzker School of Molecular Engineering, University of Chicago, Chicago, Illinois 60637, United States; ‡ Institut Charles Sadron, Université de Strasbourg, CNRS UPR22, 23 Rue du Loess, Strasbourg, 67034 Cedex 2, France; § Center for Molecular Engineering, Argonne National Laboratory, Lemont, Illinois 60439, United States

## Abstract

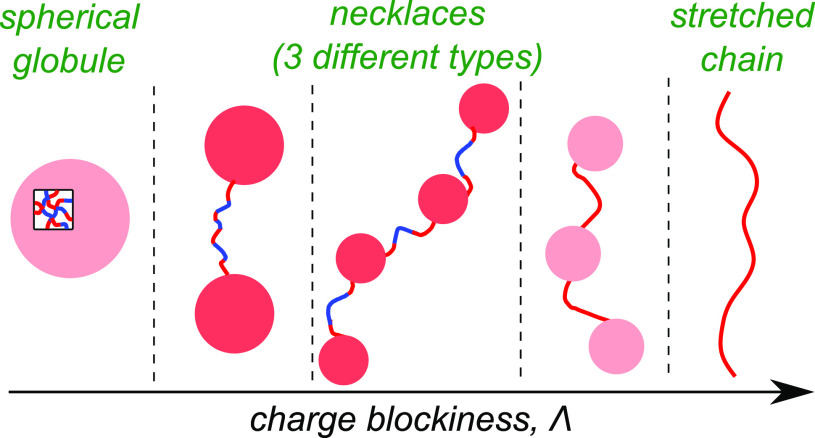

A scaling theory of statistical (Markov) polyampholytes is developed to understand how sequence correlations, that is, the blockiness of positive and negative charges, influences conformational behavior. An increase in the charge patchiness leads to stronger correlation attractions between oppositely charged monomers, but simultaneously, it creates a higher charge imbalance in the polyampholyte. A competition between effective short-range attractions and long-range Coulomb repulsions induces globular, pearl-necklace, or fully stretched chain conformations, depending on the average length of the block of like charges. The necklace structure and the underlying distribution of the net charge are also controlled by the sequence. Sufficiently long blocks allow for charge migration from globular beads (pearls) to strings, thereby providing a nonmonotonic change in the number of necklace beads as the blockiness increases. The sequence-dependent structure of polyampholyte necklaces is confirmed by molecular dynamics simulations. The findings presented here provide a framework for understanding the sequence-encoded conformations of synthetic polyampholytes and intrinsically disordered proteins (IDPs).

Polyampholytes, which carry positive and negative charges, are often viewed as synthetic analogs of proteins. There is considerable interest in understanding their conformational behaviors and their phase behavior in solution, which is relevant in a wide range of contexts, from the formation of biological subcellular compartments to the design of underwater adhesives.^1,2^ Theoretical advances in statistical physics^2−6^ have been successful in describing a number of important features of intrinsically disordered proteins (IDPs),^7−9^ a class of proteins that lack a stable secondary and tertiary structure and that undergo pronounced size and shape fluctuations.^10,11^

IDPs are a pervasive component of the membraneless organelles (MOs) that exist within living cells;^11^ the macroscopic phase separation (or “self-coacervation”^12^) that arises in PA solutions represents a reasonable model with which to study their formation.^13,14^ It is now known that the extensive clustering of opposite charges in the primary structure of IPDs facilitates the spontaneous self-assembly of MOs in vivo and in vitro.^15^ The available experimental results have been theoretically rationalized, first^16^ within the random phase approximation (RPA) following the method of ref (4) and, more recently, using other theoretical and simulation approaches.^17−19^

Less is known about the effects of sequence and charge distribution or statistics on the single-chain conformations of PAs and IDPs.^20^ Alternating PAs have zero net charge and, if sufficiently long, form globules.^4,21−23^ However, PAs with an ideal random distribution of positively and negatively charged monomers can adopt a necklace-like conformation due to the statistical deviations of the global PA charge from the ensemble-average zero value.^2,22,24−28^ Pearl-necklace conformations are often encountered in hydrophobic polyelectrolytes (PEs). They were first predicted theoretically and in simulations^29−31^ and later observed in experiments.^32−35^ Necklace formation in PAs and hydrophobic PEs is the manifestation of the Rayleigh instability^36^ of the charged spherical (or elongated cylindrical^30,37^) globule. The only difference is that, in PEs, the attractive interactions that facilitate globular conformations have a hydrophobic nature, whereas in PAs they are due to sequence-dependent Coulomb attractions between opposite charges.

In vivo, the mechanism that maintains circadian rhythms is based on the Rayleigh instability of special IDPs, which serve as a molecular hourglass.^10,38^ Their continuous phosphorylation lasts many hours, until the increasing net charge leads to a conformational transition within them. The latter triggers a set of biochemical processes that results in the reset of the circadian clocks.^38^

In this work, we develop a scaling theory of statistically neutral Markov PAs and demonstrate that PA sequence controls the conformations of the molecules, including necklace formation and structure. We consider flexible PAs containing *N* monomers, each with size *a*. A fraction *f* of the monomers is ionic; they are spaced equidistantly and each carries a charge ±*e*. The quenched sequence of positive and negative charges obeys first-order Markov process statistics with correlation parameter^39,40^
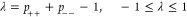
1Here *p*_*ij*_ = *p*(*i*|*j*) is the conditional probability to have an ionic monomer of type *i* after one of type *j* (*i*, *j* = +, – ), and *p*_++_ = *p*_––_ maintains the statistical neutrality of the molecules. By setting λ = −1 and 0, one recovers alternating and ideally random statistics of charges, respectively. The limiting case of λ = 1 corresponds to a stoichiometric mixture of polyanions and polycations.^40^ The average charge of the block of consecutive like charges is 1 + Λ with^23,41^
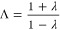
2At λ > 0, each block contains (1 + Λ)/*f* ≃ Λ/*f* monomers, and each PA consists of about  charge blocks. Due to the statistical independence of the charge signs, the average global PA charge (absolute value in *e* units) is^23,41^
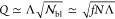
3Note that this result is valid for any λ.^41^ PAs are immersed in a salt-free Θ solvent with dielectric constant ϵ, and *u* = *l*_B_/*a* = *e*^2^/ϵ*ak*_B_*T* is its dimensionless Bjerrum length. A more detailed description of the PA model can be found elsewhere.^23,41^

We start our analysis with globally neutral PAs, *Q* = 0 (see also ref (23)). They form spherical globules whose dimensions are controlled by the statistics of charges and decrease as the charge blockiness Λ increases. In brief, an alternating PA (Λ = 0) can be considered as a chain of *Nf*/2 connected dipoles, each with dipole moment *p* ≃ *af*^–1/2^, owing to the locally Gaussian chain conformations within the globule. The energy of (Keesom) pairwise interactions between the permanent dipoles is given by *W*/*k*_B_*T* ≃ −*l*_B_^2^*p*^4^/*r*^6^, and the corresponding second virial coefficient is given by *B*_dip_ ≃ −*a*^2^*u*^2^*d* ≃ −*a*^3^*u*^2^*f*^–1/2^. Dipole–dipole attractions are balanced by three-body repulsions between all PA monomers, *B*_dip_*n*_nip_^2^ + *Cn*^3^ = 0, where *C* ≃ *a*^6^ is the third virial coefficient; *n* and *n*_dip_ ≃ *fn* are the concentrations of all monomers and dipoles. This yields the equilibrium polymer volume fraction (density) of the globule formed by alternating PA^4,23^
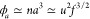
4and the correlation length (mesh size, concentration blob) in it, ξ_*a*_ ≃ *a*ϕ_*a*_^–1^ ≃ *au*^–2^*f*^–3/2^.

As Λ increases and the charge statistics gradually changes from alternating to ideally random (Λ = 1), each concentration blob ceases to be electrically neutral, as shown in Figure 1. Its charge equals , with *g* ≃ (ξ/*a*)^2^ being the number of monomers per blob. The interior of the PA globule is a correlated melt of oppositely charged blobs: The closest neighbors of the positively charged blob predominantly have a negative charge and vice versa.^2^ The energy of Coulomb correlation attractions per blob equals *F*_attr_/*k*_B_*T* ≃ *l*_B_*q*^2^/ξ, and it is balanced by three-body repulsions with the energy *F*_rep_/*k*_B_*T* ≃ ξ^3^*Cn*^3^ ≃ 1. The resulting blob size is given by ξ_*r*_ ≃ *a*/*uf*Λ and the globule density reads^23^
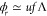
5

**Figure 1:**
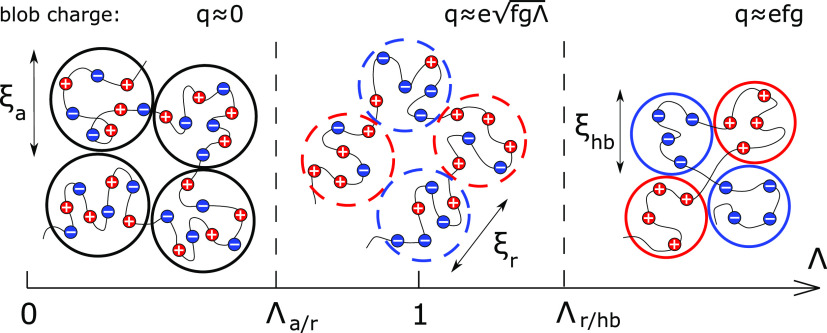
Internal structure (blob picture) of globules formed from globally neutral PAs as a function of the charge blockiness Λ. Three scaling regimes correspond to (i) almost alternating sequences, Λ < Λ_a/r_; (ii) substantially random sequences, Λ_a/r_ < Λ < Λ_r/hb_; (iii) highly blocky sequences, Λ > Λ_r/hb_.

The latter increases with increasing sequence correlations as soon as Λ exceeds
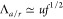
6which serves to define the crossover between alternating and substantially random sequences.^23^

This picture of the globule formed by essentially random PA holds until all charged monomers within the concentration blob are of the same sign, that is, until the block charge Λ is on the order of their number, *fg*_r_ ≃ *f*ξ_r_^2^/*a*^2^. The crossover between essentially random and highly blocky sequences is defined by^23^
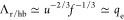
7and *q*_e_ is the charge of the electrostatic blob.^42−44^ At Λ > Λ_r/hb_, the charge of the concentration blob is equal to *q* ≃ *fg*, and the balance between Coulomb attractions and short-range repulsions, *F*_attr_ ≃ *F*_rep_, results in the globule density^23^
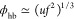
8independent of Λ. The size of the concentration blob is equal to that of the electrostatic blob, ξ_hb_ ≃ *a*(*uf*^2^)^−1/3^ ≃ ξ_e_. In other words, for PA sequences with high charge blockiness, the globule interior is similar to that of a polyelectrolyte complex coacervate,^45,46^ as seen in Figure 1 and was first proposed for the limiting case of diblock PAs.^47^

We are now in a position to consider the instability of the spherical PA globule with respect to the necklace formation induced by its nonzero charge, *Q* ≠ 0. According to Rayleigh’s criterion,^29,36^ the spherical globule splits into the smaller globules (beads or pearls) connected by the stretched chain fragments (strings) if the energy of repulsions between the excess charges *F*_Coul_^glob^/*k*_B_*T* ≃ *l*_B_*Q*^2^/*R* exceeds the surface energy of the globule *F*_surf_^glob^. The characteristic global charge of statistically neutral Markov PAs equals  and increases from *Q* = 0 for alternating sequences, Λ = 0, to *Q* ≃ *fN* for block lengths comparable to the chain length, Λ ≃ *fN*.^41^ The globule radius equals *R*_glob_ ≃ *a*(*N*/ϕ)^1/3^, and its density ϕ is given by eqs 4, 5, and 8 for the different classes of sequences (ranges of Λ). The origin of the globule surface tension is the lower number of Coulomb attractions with oppositely charged neighbors experienced by any interfacial blob. The corresponding free energy equals *F*_surf_^glob^/*k*_B_*T* ≃ *R*^2^/ξ^2^ with ξ ≃ *a*/ϕ.

The ratio *I* between the free energies that destabilize and stabilize the spherical globule
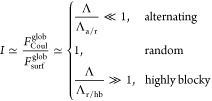
9depends on the PA statistics, Λ. Equation 9 shows that (i) for sequences close to alternating, Λ ≪ Λ_a/r_, the global charge of the PAs is low and the spherical globule is stable; (ii) for random sequences, Λ_a/r_ ≪ Λ ≪ Λ_r/hb_, (some) globules split into several beads; (iii) for highly blocky sequences, Λ ≫ Λ_r/hb_, the formation of a necklace consisting of a large number of beads is expected.

Below we consider the conformations of Markov PAs over the entire range of charge blockiness, Λ ≥ 0 (i.e., −1 ≤ λ ≤ 1), and delineate five conformational regimes, including three corresponding to pearl-necklaces of various types.

## Spherical Globule (Regime I)

At Λ ≪ Λ_a/r_, PA statistics are close to alternating, and the global charge is not sufficient to perturb the spherical globule of the radius
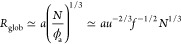
10

## Necklace with a Charge in Beads and *N*_bead_ ≃ 1 (Regime II)

For substantially random sequences, Λ_a/r_ ≪ Λ ≪ Λ_r/hb_, the global charge of the PA is close to the threshold value that triggers necklace formation (see eq 9). Different realizations of the Markov process correspond to PAs with different global charges. For long chains, some *N*-independent fractions of PAs retain a spherical shape, while another finite fraction forms necklaces.^22,24,25,41^ Since the size of the necklace far exceeds that of the spherical globule, the former provide the dominant contribution to the ensemble average dimensions of the PAs.^24^

The Rayleigh criterion 9 defines the number of monomers in the bead,^29^*m*_bead_ ≃ *N*. The necklace consists of several beads
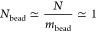
11each with radius
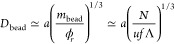
12The length of the string *l*_str_ is controlled by the balance between the Coulomb energy arising from bead repulsions *F*_Coul_^bead–rep^ and the excess surface energy of the string *F*_surf_^str^:
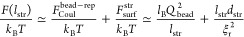
13The string thickness *d*_str_ ≃ ξ_r_ provides the energy of its elastic stretching *F*_el_^str^/*k*_B_*T* ≃ *l*_str_ξ_r_/*d*_str_^2^ that is comparable with the surface energy *F*_surf_^str^.^29^ Using  one can find the string length
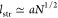
14that minimizes the free energy 13. Each string contains *m*_str_ ≃ *l*_str_ξ_r_ ≃ *N*^1/2^/*uf*Λ monomers and *m*_str_ ≪ *m*_bead_.^29^ The necklace length^2,22^
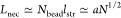
15coincides with the ideal-coil radius of the chain. For the particular case of ideally random PAs, Λ = 1, eq 15 has been confirmed in simulations.^24^

The independence of the necklace structure (*N*_bead_ ≃ 1) and length on the charge statistics Λ in Regime II is remarkable and stems from the exact compensation of two opposing tendencies. As the charge blockiness increases, Coulomb correlation attractions get stronger, ϕ_r_ ∼ Λ, thereby providing a higher surface tension of the globule/bead and a higher stability against splitting. Simultaneously, a decreasing globule/bead size (eq 12) and the increasing PA charge, , encourage Rayleigh instability. Together, this yields the value *I*(Λ) ≃ 1 found in eq 9.

## Necklace with a Charge in Beads and *N*_bead_ ≫ 1 (Regime III)

In the regime of high charge blockiness, Λ ≫ Λ_r/hb_, the strength of correlation attractions and, hence, the bead density level off (eq 8), while the global charge of the chain keeps growing. Therefore, the number of beads in the necklace *N*_bead_ and the necklace length *L*_nec_ increase with increasing Λ. The equality between the Coulomb self-energy
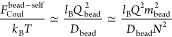
16and the surface free energy of the bead
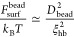
17with ξ_bh_ ≃ *a*/ϕ_hb_ defines its size
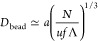
18The higher the charge patchiness, the lower the number of monomers per bead:
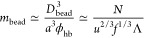
19The total number of beads (and strings) in the necklace increases with Λ linearly, from about unity at the crossover II/III:
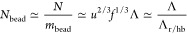
20The long-range Coulomb repulsions between beads, *F*_Coul_^bead–rep^/*k*_B_*T* ≃ *l*_B_*Q*_bead_^2^/*l*_str_, are balanced by the elasticity of the stretched string, *F*_el_^str^/*k*_B_*T* ≃ *F*_surf_^str^/*k*_B_*T* ≃ *l*_str_/ξ_hb_, and the string length is given by
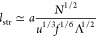
21The string thickness *d*_str_ ≃ ξ_hb_ ≃ ξ_e_ is equal to the electrostatic blob size. The number of monomers in the string is *m*_str_ ≃ *N*^1/2^/*u*^2/3^*f*^5/6^Λ^1/2^. Contrary to Regime II of random sequences, the necklace total length
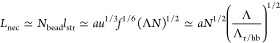
22is much larger than the Gaussian coil size.

In Regimes II and III, most of the necklace mass is concentrated in the beads, owing to *m*_str_ ≪ *m*_bead_. The distribution of the global charge almost coincides with the mass distribution, and the charge of the string is small as compared to the bead charge, *Q*_str_ ≪ *Q*_bead_. In this respect, PA necklaces II and III are similar to the necklaces of regular quenched hydrophobic PEs,^29^ where the charge is homogeneously smeared throughout the chain. However, in Markov PAs the charge distribution is patchy and the net charge is provided by just  blocks, while the total number of blocks in the chain, , is much higher. At increasing blockiness Λ, these frozen sequence fluctuations allow for the net charge redistribution from beads to strings via PA refolding. Bead-to-string migration^48^ of the net charge diminishes the Coulomb energy of the necklace because *D*_bead_ ≪ *l*_str_ and the Coulomb self-energy of the string is lower than that of the bead. Already in Regime II, migration may result in the string charge , far exceeding the mean charge of *m*_str_ monomers, . In Regime III, the increase in *Q*_str_ and net charge redistribution continue. The boundary III/IV between the necklaces with the most net charge concentrated in beads/string arises when their charges are commensurate, *Q*_str_ ≃ *Q*_bead_, and is given by
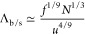
23At Λ ≃ Λ_b/s_, the bead charge, the block charge, and the number of ionic monomers in the string are all equal to each other, *Q*/*N*_bead_ ≃ Λ ≃ *fm*_str_. Boundary III/IV, given by eq 23, can also be derived via a free energy analysis, as demonstrated in the Supporting Information. We emphasize that the net charge migration is due to its inhomogeneous, blocky distribution in the Markov PAs and cannot take place in necklaces of regular quenched hydrophobic PEs.

## Necklace with Charge in Strings (Regime IV)

At Λ ≫ Λ_b/s_, the global charge of the necklace is due to the strings; beads carry a much lower charge and tend to merge with each other to diminish the surface energy. This facilitates the formation of long strings, and the string length is limited by the block length. Each string is essentially a PE containing *m*_str_ ≃ Λ/*f* monomers that adopts a stretched conformation^42−44^ with *d*_str_ ≃ ξ_e_ and
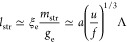
24Here *g*_e_ ≃ ξ_e_^2^/*a*^2^ ≃ (*uf*^2^)^−2/3^ is the number of monomers in the electrostatic blob. The number of the necklace beads
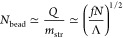
25decreases with increasing Λ. Each bead comprises
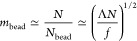
26monomers and its size equals
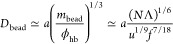
27The length of the new type of necklace
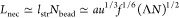
28obeys the same scaling law as that of the usual necklace in Regime III, eqs 22. However, these necklaces have different structures, as seen from eqs 20 and 25, which define the number of beads.

We note that, in Regime IV, a minor fraction of the necklace charge is still located in the beads. Using the equality between the electrostatic potentials of the bead and the string,^47^*Q*_bead_/*D*_bead_ ≃ *Q*_str_/*l*_str_, one can find the bead charge
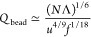
29which is much lower than *Q*_str_ ≃ Λ.

The crossover to the PE regime occurs at
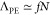
30when the block length Λ/*f* and the chain length *N* become comparable. At Λ ≃ Λ_PE_, when the PAs comprise several charge blocks, eq 25 predicts *N*_bead_ ≃ 1 and the necklace length *L*_nec_ ≃ *au*^1/3^*f*^2/3^*N* given by eq 28 coincides with the PE chain size. This is consistent with the tadpole conformations predicted for length-asymmetric non-neutral diblock PAs (block lengths *N*_+_ ≠ *N*_–_).^47^ Each tadpole consists of an almost neutral globular head and an extended PE tail (or two tails) carrying almost the entire net charge of the PA.^47^ At |*N*_+_ – *N*_–_| ≃ *N*_+_ ≃ *N*, the head size and charge found in ref (47) coincide with the results of eqs 27 and 29 for crossover IV/V given by Λ ≃ Λ_PE_: *D*_bead_ ≃ *au*^–1/9^*f*^–2/9^*N*^1/3^ and *Q*_head_ ≃ *u*^–4/9^*f*^1/9^*N*^1/3^. In the language of ref (47), necklace IV with the charge in the strings is the set of the jointed PA tadpoles.

## Stretched Polyelectrolytes (Regime V)

If the block is much longer than the chain, Λ ≫ Λ_PE_, the probability of each PA carrying only positive/negative charges is *e*^–Λ_PE_/Λ^. It is essentially a PE of length^42−44^
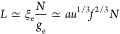
31The statistical neutrality of the PAs is manifest in the equal number of polyanions and polycations in the ensemble.^40^

**Figure 2:**
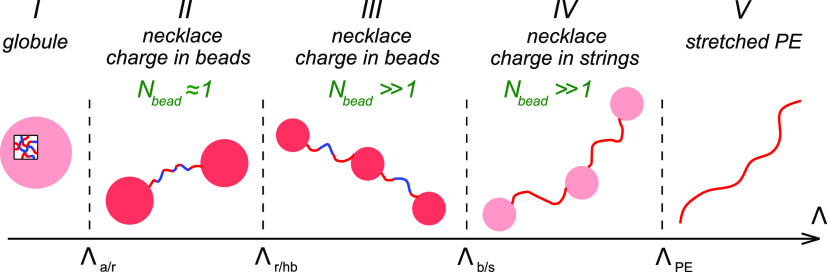
Schematic representation of PA conformations in different conformational regimes. The color of the globular beads represents their non-neutralized charge. The charge of the red beads is about the critical value triggering their Rayleigh instability, and that of the pink beads is much below the critical threshold.

**Figure 3:**
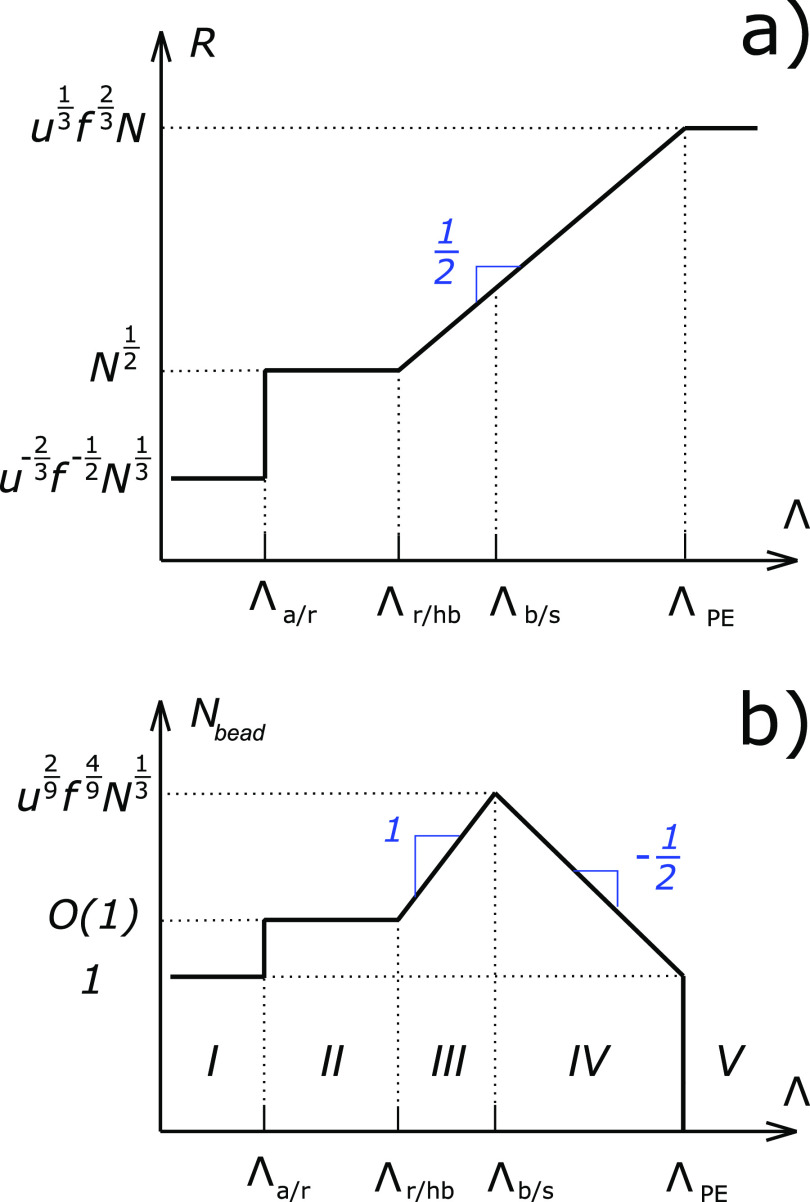
Dependencies of (a) the ensemble average radius of gyration *R* and (b) the number of the globular beads *N*_bead_ on the charge blockiness Λ.

The results of our scaling analysis are summarized in Table 1 and Figure 2, which provide illustrative conformations of Markov PAs in different regimes. Figure 3a represents the dependence of the ensemble average dimensions of the PAs on the charge blockiness, *R* versus Λ. For PAs having sequences (Markov process realizations) providing the dominant contribution to *R*, the number of globular beads *N*_bead_ changes in a nonmonotonic fashion and reaches a maximum at Λ ≃ Λ_b/s_, as seen in Figure 3b.

**Table 1:** Scaling Laws for Conformational Properties of PAs: Chain Radius of Gyration, *R*; Number of Beads, *N*_bead_; Number of Monomers in Bead, *m*_bead_, and Strings, *m*_str_; Bead Size, *D*_bead_; String Length, *l*_str_^a^

	*R*/*a*	*N*_bead_	*m*_bead_	*m*_str_	*D*_bead_/*a*	*l*_str_/*a*
*I*	*N*^1/3^/*u*^2/3^*f*^1/2^	=1	*=N*		*N*^1/3^/*u*^2/3^*f*^1/2^	
*II*	*N*^1/2^	1	*N*	*N*^1/2^/*uf*Λ	(*N*/*uf*Λ)^1/3^	*N*^1/2^
*III*	*u*^1/3^*f*^1/6^(*N*Λ)^1/2^	*u*^2/3^*f*^1/3^Λ	*N*/*u*^2/3^*f*^1/3^Λ	*N*^1/2^/*u*^2/3^*f*^5/6^Λ^1/2^	(*N*/*uf*Λ)^1/3^	*N*^1/2^/*u*^1/3^*f*^1/6^Λ^1/2^
*IV*	*u*^1/3^*f*^1/6^(*N*Λ)^1/2^	(*fN*/Λ)^1/2^	(*N*Λ/*f*)^1/2^	Λ/*f*	(*N*Λ)^1/6^/*u*^1/9^*f*^7/18^	(*u*/*f*)^1/3^Λ
*V*	*u*^1/3^*f*^2/3^*N*	0		*=N*		*u*^1/3^*f*^2/3^*N*

a Globules and stretched PEs consist of 1 bead and 1 string, respectively.

In our analysis, we have primarily adopted a mean-field approach that neglects quenched charge fluctuations, except in regime IV, where blockiness is essential. At the III/IV transition, the bead charge is found to drop by a factor (*N*/*g*_e_)^1/9^, while other quantities remain continuous. Our results predict ensemble-averaged properties of PAs, while the effect of the sequence realization, that is, quenched (frozen) disorder,^27,28,39,40,49−53^ on their conformation remains to be explored. Focused numerical simulations^24,25^ covering the vicinity of this transition would help clarify how sequence disorder smears it out, and affects the cooperativity expected from the Landau theorem.^54^ How quenched sequences under thermal agitation fall (statistically) in Regime III or IV and whether both structures can follow each other along the same sequence of a reasonable length are of particular interest.

**Figure 4:**
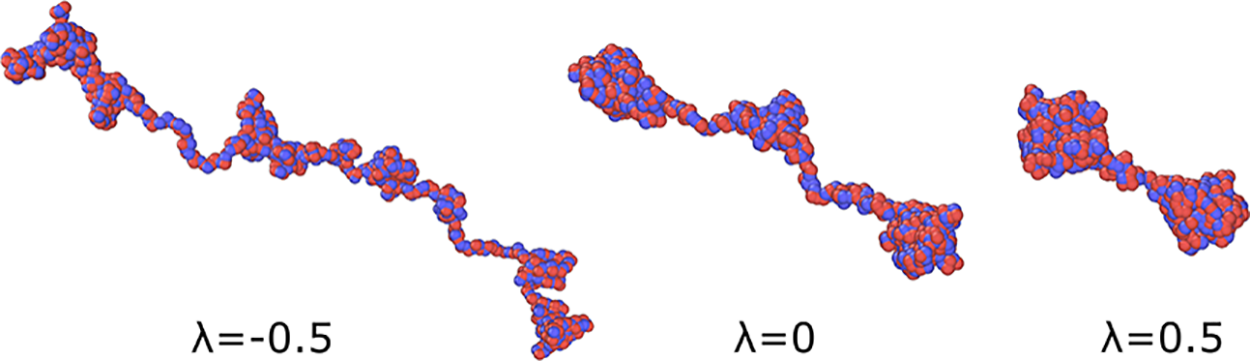
Molecular dynamics simulations of Markov PAs with fixed global charge, *Q* = 40, and different charge statistics, λ. The ensemble-averaged gyration radii of PAs with *N* = 1024 and *f* = 1 are equal to *R*_g_ = 31.6 ± 3.6 for λ = −0.5, *R*_g_ = 20.9 ± 2.4 for λ = 0, and *R*_g_ = 10.9 ± 2.3 for λ = 0.5. The PA conformations shown here illustrate that the increasing charge blockiness leads to more compact PA conformations. Simulation details can be found in ref (23) and the SI.

The set of predicted conformational changes with increasing Λ is a consequence of two competing tendencies: an increase in Coulomb correlation attractions and in the global charge imbalance of a single PA. As an example, one can consider the series of PAs having increasing charge blockiness, but constant global charge so as to keep operative only the former factor. Figure 4 shows that Markov PAs with fixed global charge become more compact as the charge clustering increases. The observation of different PA conformations/dimensions along a similar set of (e.g., sequence-monodisperse) globally charged PAs could provide a simple way to verify our predictions in laboratory experiments.

In conclusion, the sequence specificity of Markov PA conformations has been analyzed, and five conformational regimes have been delineated. The boundaries between these regimes are controlled by the interplay between the inherent sequence scales and the characteristic physical lengths of the problem.^55^ When the block charge exceeds that of the electrostatic blob, Λ > *q*_e_, PA necklaces start to stretch, and the number of beads *N*_bead_, increases (crossover II/III). The equality between the block and the bead charges defines boundary III/IV and is accompanied by the migration of the non-neutralized charge from necklace beads to strings and by an *N*_bead_ decrease with increasing charge blockiness.

## Supporting Information Available

(1) Boundary III/IV between the necklaces with the net charge in beads and strings: Comparison of the free energies; (2) The details of molecular dynamics simulations (PDF)
