# Supplementary material for: Sequence Blockiness Controls the Structure of Polyampholyte Necklaces
Source: ACS Macro Lett. 2021 Jul 28;10(8):1048–54. doi: 10.1021/acsmacrolett.1c00318 (PMC8397398; doi:10.1021/acsmacrolett.1c00318)
Supplement: Supplementary file 1 — mz1c00318_si_001.pdf [file mz1c00318_si_001.pdf]

Supporting Information

Sequence Blockiness Controls the Structure of Polyampholyte Necklaces

Artem M. Rumyantsev,[†] Albert Johner,[*,‡] and Juan J. de Pablo[*,†,¶]

†*Pritzker School of Molecular Engineering, University of Chicago, Chicago, Illinois 60637, United States*

‡*Institut Charles Sadron, Université de Strasbourg, CNRS UPR22, 23 Rue du Loess, Strasbourg 67034 Cedex 2, France*

¶*Center for Molecular Engineering, Argonne National Laboratory, Lemont, Illinois 60439, United States*

E-mail: albert.johner@ics-cnrs.unistra.fr; depablo@uchicago.edu

1. Boundary III/IV between the necklaces with the net charge in beads and strings: Comparison of the free energies

The described structure of both types of necklaces, with charge in beads III and charge in strings IV, allows for deriving the boundary III/IV between them

$$\Lambda_{b/s} \simeq \frac{f^{1/9} N^{1/3}}{u^{4/9}} \tag{S1}$$

using the free energy comparison. If necklace IV with the charged strings also forms below the boundary, at $\Lambda < \Lambda_{b/s}$, it would have a higher number of beads as compared to necklace III with the charged beads. This results in the excess surface energy

$$\frac{F_{bead}^{surf}}{k_B T} \simeq \frac{D_{bead}^2}{\xi_{hb}^2} \simeq u^{4/9} f^{5/9} (N\Lambda)^{1/3} \tag{S2}$$

per bead and $N_{bead} F_{bead}^{surf} \sim \Lambda^{-1/6}$ per PA chain. Here we used D_{bead} and N_{bead} found for necklace IV and given by eqs. 27 and 25. The energy gain in necklace IV is provided by negligible Coulomb self-energy of beads, which for necklace III is given by eq. 16 and equals

$$\frac{F_{Coul}^{bead-self}}{k_B T} \simeq \left(\frac{fN}{\Lambda}\right)^{2/3} \tag{S3}$$

per bead and $N_{bead} F_{Coul}^{bead-self} \sim \Lambda^{1/3}$ per PA. Thus, at $\Lambda \leq \Lambda_{b/s}$, the transition between necklaces III and IV is accompanied by the Coulomb energy gain $\sim \Lambda^{1/3}$ but the surface energy cost $\sim \Lambda^{-1/6}$ and therefore takes place at increasing Λ. Owing to the continuously changing N_{bead}, the balance between the energy gain and lost reads $F_{bead}^{surf} \simeq F_{Coul}^{bead-self}$, with the energies per per bead given by eqs. S2 and S3. This leads to the boundary position defined by eq. S1 and eq. 23 of the main text.

2. The details of molecular dynamics simulations

Simulations were performed using LAMMPS package[1] and following our earlier work 2. In brief, Markov PAs were modelled as the particles connected by finitely extensible nonlinear elastic (FENE) springs

$$U_{FENE}(r) = -\frac{1}{2}KR_0^2 \ln\left(1 - \frac{r^2}{R_0^2}\right) \tag{S4}$$

and interacting via truncated and shifted Lennard-Jones potential

$$U_{LJ}(r) = \begin{cases} 4\varepsilon_{LJ}\left[\left(\frac{\sigma}{r}\right)^{12} - \left(\frac{\sigma}{r}\right)^{6} - c(R_c)\right], & r \le R_c \\ 0, & r \ge R_c \end{cases} \tag{S5}$$

with $c(R_c) = (\sigma/R_c)^{12} - (\sigma/R_c)^6$. The values of the parameters $\varepsilon_{LJ} = 0.34k_BT$, $R_c = 2.5\sigma$, $K = 7k_BT/\sigma^2$, and $R_0 = 2\sigma$ were chosen to provide Θ solvent conditions.[3,4] Ionic monomers interact with each other via a bare Coulomb potential

$$U_{Coul}(r) = k_BT\frac{l_b z_i z_j}{r_{ij}} \tag{S6}$$

with $z_{i,j} = \pm 1$ being their charge valence and r_{ij} being the distance between these monomers. Bjerrum length is set to $l_b/\sigma = 3$, and solvent is considered implicitly, as the continuous dielectric medium.

Sequences of PAs with $f = 1$ were generated from the Markov process with the correlation parameter values, $\lambda = -0.5$, 0, and 0.5. For each λ, these sequences provide the ensemble of statistically neutral PAs, i.e., the ensemble-averaged charge of these PAs is zero. Then, the sequences (Markov process realizations) providing the required nonzero global charge of the PA, Q, were chosen to perform further simulations. To provide reliable averaging over the sequence realizations, simulations were performed for $\mathcal{N}$ PAs with different sequences. $\mathcal{N} = 10$ was sufficient to obtain the average PA gyration radius independent of the given sequence realizations.

The time step for simulations was equal to $0.005\tau_{LJ}$, and the temperature of the system, $T = 1$, was maintained by the Langevin thermostat, and the damping factor was equal to damp $= 100\tau_{LJ}$.[2] For each PA realization, the MD run consisted of 5×10^6 equilibration steps ($2.5 \times 10^4 \tau_{LJ}$) followed by 1.5×10^7 productions steps ($7.5 \times 10^4 \tau_{LJ}$) to get the PA gyration radius, R_g. The equilibrium sampling is ensured by the analysis of the autocorrelation function of R_g.[2,4] For $N = 256$, the relaxation times were in the range of $(1 - 3) \times 10^4$ steps, i.e., $(50 - 150)\tau_{LJ}$, depending on the sequence and charge blockiness, λ. For $N = 1024$, the relaxation times were estimated to be $(3-9)\times 10^4$ steps, i.e., $(150-450)\tau_{LJ}$. Higher relaxation times were observed for higher λ values. We emphasize that the relaxation time is strongly dependent on the sequence realization, and our estimates correspond to the characteristic ensemble-averaged values. The necklace structures were found to be strongly fluctuating. During the simulation run, PAs switched between conformations with the different number of beads many times, thereby confirming the reliability of our averaging.[5,6]

For $N = 256$ and $N = 1024$, the global charge value was chosen to be $Q = 20$ and $Q = 40$, respectively. These charge values are on the order of the characteristic global charge (absolute value) of Makrov statistically neutral PAs, $\sqrt{fN\Lambda}$, and therefore do not strongly bias their charge statistics.[7] The resulting values of R_g are summarized in Table S1 and conclusively demonstrate that PA conformations are strongly sequence-dependent. For both short and long PAs, their dimensions decrease at increasing charge blockiness, λ.

Table S1: Gyration radius of Markov PAs containing N monomers and carrying fixed nonzero global charge Q.

	$\lambda = -0.5$	$\lambda = 0$	$\lambda = 0.5$
$N = 256$ and $Q = 20$	13.5 ± 1.8	8.2 ± 2.0	5.8 ± 0.8
$N = 1024$ and $Q = 40$	31.6 ± 3.6	20.9 ± 2.4	10.9 ± 2.3

References

(1) Plimpton, S. Fast parallel algorithms for short-range molecular dynamics. J. Comput. Phys. 1995, 117, 1–19.

(2) Rumyantsev, A. M.; Jackson, N. E.; Johner, A.; de Pablo, J. J. Scaling theory of neutral sequence-specific polyampholytes. *Macromolecules* **2021**, 54, 3232–3246.

(3) Micka, U.; Holm, C.; Kremer, K. Strongly charged, flexible polyelectrolytes in poor solvents: Molecular dynamics simulations. *Langmuir* **1999**, 15, 4033–4044.

(4) Wang, Z. ; Rubinstein M. Regimes of conformational transitions of a diblock polyampholyte. *Macromolecules* **2006**, 39, 5897–5912.

(5) Yamakov, V.; Milchev, A.; Limbach, J. H.; Dünweg, B.; Everaers, R. Conformations of random polyampholytes. *Phys. Rev. Lett.* **2000**, 85, 4305–4308.

(6) Lee, N.-K.; Jung, Y.; Johner, A.; Joanny, J.-F. Globular polyampholytes: Structure and translocation. *Macromolecules* **2021**, 54, 2394–2411.

(7) Johner, A.; Joanny, J.-F. Translocation of polyampholytes and intrinsically disordered proteins. *Eur. Phys. J. E* **2018**, 41, 78.
